# Supplementary material for: Inflammatory factors and risk of lung adenocarcinoma: a Mendelian randomization study mediated by blood metabolites
Source: Front Endocrinol (Lausanne). 2024 Aug 27;15:1446863. doi: 10.3389/fendo.2024.1446863 (PMC11384989; doi:10.3389/fendo.2024.1446863)
Supplement: Supplementary file 1 [file DataSheet1.zip › supplementary files/S2/GCST90200274/forest.pdf]

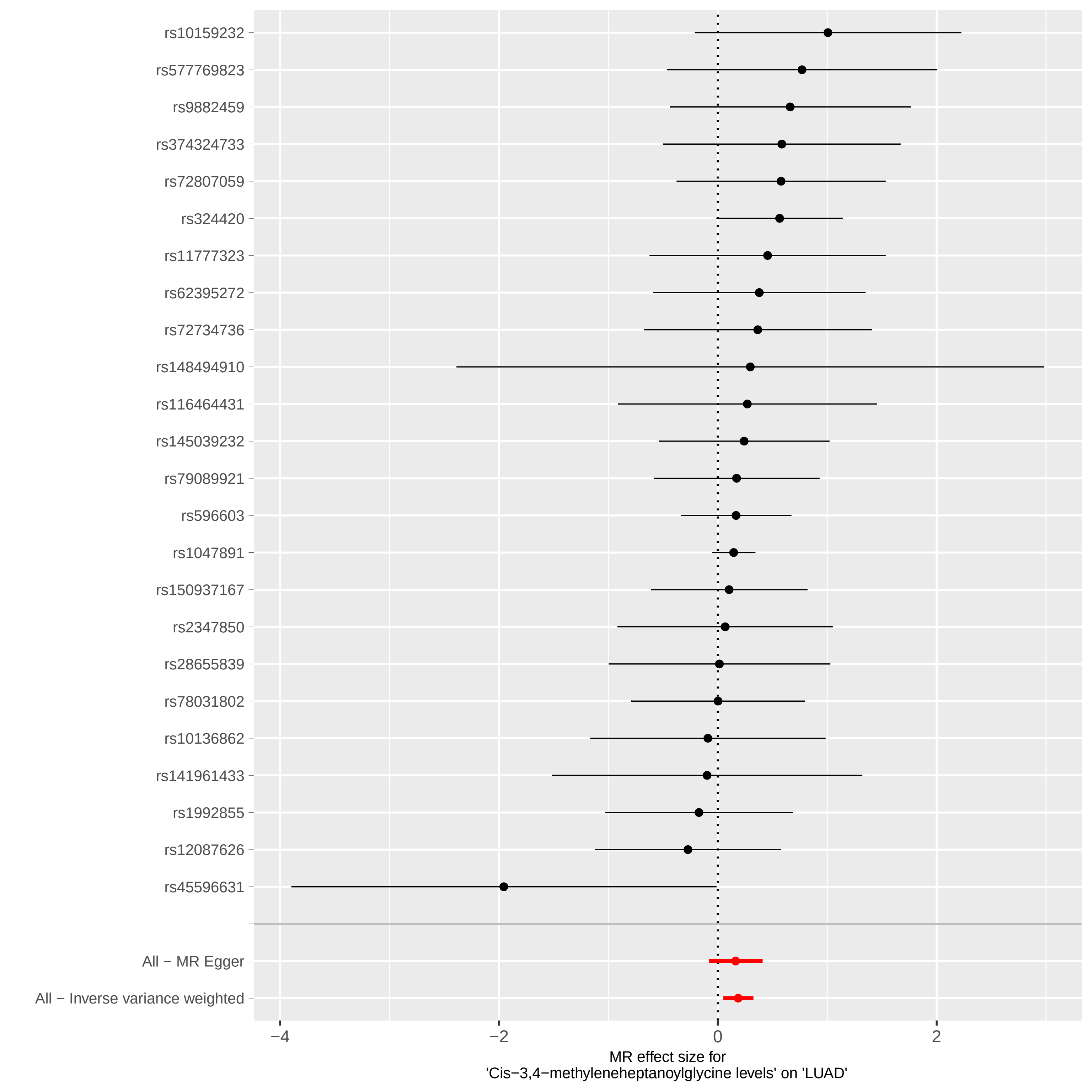
rs10159232
rs577769823
rs9882459
rs374324733
rs72807059
rs324420
rs11777323
rs62395272
rs72734736
rs148494910
rs116464431
rs145039232
rs79089921
rs596603
rs1047891
rs150937167
rs2347850
rs28655839
rs78031802
rs10136862
rs141961433
rs1992855
rs12087626
rs45596631
All − MR Egger
All − Inverse variance weighted
−4
−2
0
2
MR effect size for
'Cis−3,4−methyleneheptanoylglycine levels' on 'LUAD'
